# Hemorrhage in a Fetal Ovarian Cyst

**DOI:** 10.21699/jns.v6i2.505

**Published:** 2017-04-15

**Authors:** RAA Hassan, YC Chong, M Khairun Nisa, CG Yew, PG Tan, JAN Mazlin

**Affiliations:** 1 Department of Surgery, School of Medical Science, Universiti Sains Malaysia, Kelantan, Malaysia; 2 Department of Obstetrics and Gynaecology, School of Medical Science, Universiti Sains Malaysia, Kelantan, Malaysia

**Dear Sir**

A 38-year-old lady, gravida 4, attended antenatal check-up at 34 weeks of gestation, ultrasonography was done and the fetus noted to have an intra-abdominal cystic mass. She had no significant past medical history, and previous antenatal follow up was uneventful. The mass was hyperechoic with well-defined margin. It measures 8.2cm x 7.7cm, and occupied nearly the entire fetal abdomen. Reassessment of the mass at 35-week gestation noted increment in its size. On suspicion of intra-cystic haemorrhage and the mother’s obstetric history of multiple caesarean sections, delivery via emergency lower segment caesarean section was chosen. The baby was delivered with a good Apgar score and her birth weight was 2.65kg. She was clinically pink and not in respiratory distress despite having a grossly distended abdomen.

Further investigations found her to be anemic requiring blood transfusion. Beta-hCG level was 153.8 IU/L, and alpha-feto protein level was 995328 ug/mL. The abdominal ultrasound and CT scan showed a huge, complex cystic pelvic mass measuring 10 x 7 x 10cm (Fig.1A). Laparotomy was done on day 2 of life. Intraoperatively, she had a huge left ovarian cyst with intra-cystic haemorrhage (Fig.1B). The cyst was twisted and adherent to the left fallopian tube. Left salphingoopherectomy was done. The right ovary and fallopian tube was normal. Histopathological examination confirmed a haemorrhagic follicular ovarian cyst with no malignant component. She had an uneventful recovery.

Neonatal ovarian cyst (OC) was first reported in 1889 by Mudholkar et al [2]. Since the routine use of prenatal ultrasound, the diagnosis of fetal ovarian cyst becomes more common [1]. Most cases of fetal OC are diagnosed in third trimester. Nussbaum et al [3] classified ultrasonic criteria of neonatal OC into simple and complex cyst. Complex cysts are associated with intracystic hemorrhage and ovarian torsion in 89% of cases, although there has been report of intracystic hemorrhage without torsion [4]. In our patient, we have found a huge complex left ovarian cyst with torsion and intra-cystic hemorrhage. Some authors have practiced intrauterine puncture and aspiration of both simple and complex cysts [5]. In our case as the baby was near term so we went for delivering the fetus for any intervention. Operative procedure should be as conservative as possible, with laparoscopic exploration preferred over open method, and viable ovary should be attempted to be preserved whenever possible. In our case, unfortunately the ovarian cyst was adhered to the fallopian tube and no viable ovary was seen hence salphingo-oophorectomy was done.

**Figure:**
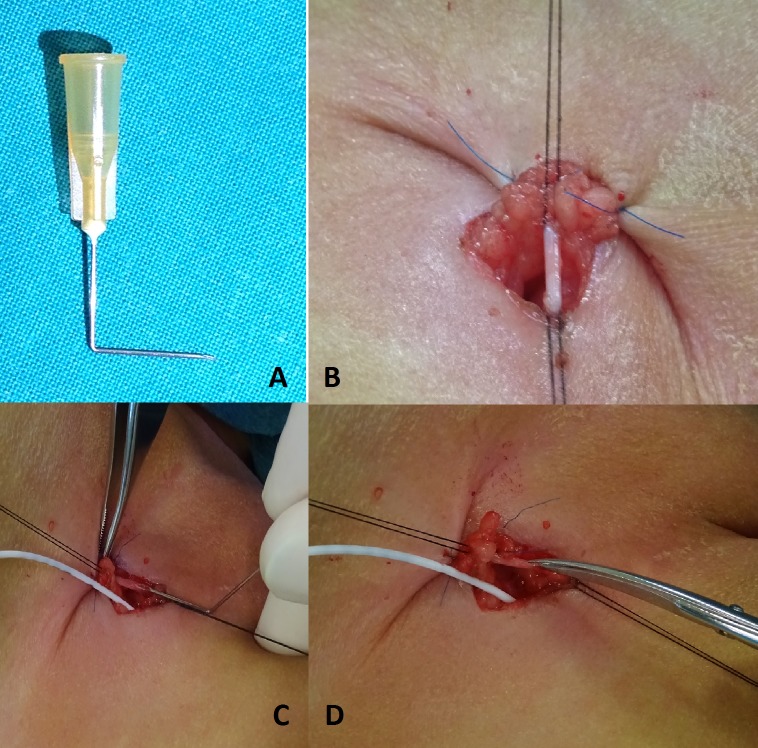
Figure 1: (A) CT scan showing huge cyst. (B) Intraoperative finding.

## Footnotes

**Source of Support:** Nil

**Conflict of Interest:** None
